# The psychological motivation of users actively constructing information cocoons from the perspective of Adler’s teleology: an empirical study based on a sample of Chinese university students

**DOI:** 10.3389/fpsyg.2026.1742055

**Published:** 2026-04-24

**Authors:** Huiyuan Wu, Yuhan Zou, Yuxi Zou

**Affiliations:** 1 School of Literature and Journalism, Guangdong Ocean University, Zhanjiang, Guangdong, China; 2 School of Law and Politics, Guangdong Ocean University, Zhanjiang, Guangdong, China

**Keywords:** active construction, Alder’s teleology, algorithm recommendation, dual systems, information cocoon, psychological motivation

## Abstract

With the advancement of internet technology, the phenomenon of information cocoons has become increasingly prominent. Existing research, focusing on algorithms and personal preferences, tends to view users as passive recipients of information, overlooking the fact that audiences in the Web3.0 era are algorithm-aware and possess autonomy in their choices. Therefore, this study adopts a new perspective by treating users as proactive agents and introduces Adler’s teleology to explore the psychological motivations behind users’ active construction and maintenance of information cocoons. The study categorizes user psychological motivations into six dimensions, revealing that sense of belonging (*B* = 0.623***), sense of control and security (*B* = 0.620***), self-consistency maintenance (*B* = 0.554***), superiority (*B* = 0.509***), goal orientation (*B* = 0.578***), and avoidance of failure and negative emotions (*B* = 0.323***) all exert significant positive effects on users’ proactive information cocooning behaviors. Subsequently, the study categorized the six psychological dimensions into two motivational systems: proactive motivation and defensive motivation. Through moderation analysis, the relationship between proactive and defensive motivation in influencing information cocooning behavior was examined, revealing an inhibitory moderation effect between the two. By examining the moderating effects of the six psychological motivations, it was found that defensive motivation and proactive motivation exerted roughly equivalent moderating strengths on users’ proactive information cocooning behaviors, with no clear priority. Both play equally important roles in influencing user behavior.

## 1 Introduction

Big data era tech advancements have intensified network dependency and selective exposure in information behaviors (Yuan and Wang, 2022). Such progress has implicitly fostered a phenomenon termed the “information cocoon” (Kar and Moinuddin, 2025). In 2023, a survey report by China Youth Daily revealed that among 1,501 Chinese respondents, 62.2% explicitly stated that the precision targeting of big data and algorithms had trapped them within information cocoons (Pinzhi and Ding, 2023).

The growing prominence of information cocoons across various domains has attracted increasing scholarly attention (Sukiennik et al., 2024; Han et al., 2022). This concept refers to the situation where the audience only selects topics they are interested in and find pleasant from a vast amount of information, while rejecting or ignoring other viewpoints and contents (Sunstein, 2006). Existing research identifies technology and personal preferences as primary drivers of information cocoon (He, 2025). Within this framework, algorithmic recommendations constitute an external stimulus, while preference-based information selection is viewed as an internal response. This “stimulus–response” (Skinner, 1965) interpretive paradigm positions users as passive, reactive audiences. However, from Web1.0 to 3.0, the identity of information recipients has evolved from “audience” to “users” (Xinglan, 2020). Users can now play an active, subject role by choosing how to interact with algorithmic systems (Hu and Ou, 2025). Yet today, the information cocoon phenomenon remains difficult to break (Zhang et al., 2023). This suggests users possess the potential to actively construct and maintain information cocoons. Based on this, we introduces Adler’s Teleology, viewing information cocoons as outcomes of users’ active information construction behaviors, and investigates the underlying purposes behind these user actions.

## 2 Literature review

### 2.1 Research status of information echo chambers

#### 2.1.1 Concept of the information cocoon

Information cocoons have emerged alongside the Web’s evolution from 1.0 to 3.0 since the internet’s 1995 rise (Du, 2024). In 2006, Professor Sunstein first proposed the concept of “information cocoon,” which refers to individuals only accessing self-aligned viewpoints due to algorithmic mechanisms and personal preferences, thus forming a closed information environment (Sunstein, 2006). This concept suggests that the formation of information cocoons is linked to users’ active selection of information based on their preferences. However, with technological advancements, an increasing number of studies lean toward technological determinism, suggesting that the maturation of algorithmic technologies has intensified the information cocoon phenomenon (Zhang et al., 2023).

#### 2.1.2 Technological causes of the information cocoon

With technological advancements, the internet era has witnessed an information explosion (Fuertes et al., 2020). With limited attention (Cinelli et al., 2020) and information overload, users often rely on algorithms to select content (Kitsuregawa and Nishida, 2010). Algorithms analyze user behavior, visualize data, and track interactions (Belo-Olgaz et al., 2016; Salihan and Kim, 2016) to filter and push information, recommending content aligned with users’ preferences and perspectives (Zhang et al., 2025; Zhou and Han, 2019). While this mechanism satisfies users’ information needs and reduces the cost of information acquisition (Yao, 2020; Wang, 2023), algorithms’ overfitting to historical interaction data directly leads to content homogenization in recommendations (Zhang et al., 2025) and constrains individuals’ information selection processes (Schmidt et al., 2017). Algorithms will shape an invisible “filter bubble” (Erickson, 2024). This filter bubble encompasses homogenized recommended content (Areeb et al., 2023), biased information dissemination (Liang et al., 2021), and ideological isolation among users (Min et al., 2019; Flaxman et al., 2016). It isolates users from diverse perspectives, often without their awareness (Geschke et al., 2019). Under the influence of long-term feedback loops, the information cocoon effect intensifies further (Mansoury et al., 2020).

#### 2.1.3 Individual contributors to the information cocoon

Beyond algorithmic recommendations, the information-seeking process is also influenced by the seeker’s existing beliefs (Johnstone, 1996). People’s information needs are inherently partial, leading individuals to unconsciously consume content that aligns with their existing cognitive frameworks through selective exposure (Kar and Moinuddin, 2025). This selectivity is termed confirmation bias (Jonas et al., 2001). Driven by cognitive preferences, users subconsciously disregard valuable information unrelated to their needs (Yuan and Wang, 2022). They also automatically avoid opposing viewpoints and seek information that may reinforce their existing attitudes and behaviors (Hart et al., 2009). That is, users interact only with information that aligns with their beliefs, failing to engage with opposing viewpoints or contradictory information (Liu et al., 2025). Users derive comfort from browsing familiar content, reducing their willingness to actively seek alternative information (Ren et al., 2021) and passively interacting with algorithmically recommended content.

Furthermore, scholars have discovered that emotional factors also dominate users’ information selection (Del Vicario et al., 2016). Emotional content more readily captures user attention, leading users to unconsciously become trapped in polarized information environments (He, 2025). This indicates that users construct information cocoons not only due to cognitive preferences but also driven by emotional factors.

### 2.2 Limitations of existing research

Existing studies have analyzed the causes of the information cocoon from the perspectives of algorithmic technology and individual preferences, but most of them follow the “causality theory” or “stimulus–response” interpretive paradigms. Under this paradigm, users are more often regarded as passive, reactive objects and are traditionally considered audiences.

However, media technology’s evolution has transformed information recipients from “audience” to “user” (Chen et al., 2016). The Web 1.0 era, termed the “read-only web,” employed a top-down “one-to-many” communication model (Kar and Moinuddin, 2025). Individuals during this period were in a passive state of receiving information (Guan and Liu, 2019), that is, the so-called audience. In contrast, the Web 2.0 era saw individuals become participatory (Kar and Moinuddin, 2025). The term “user” has been introduced into the new media ecosystem (Yang and Liu, 2017) and is gradually replacing “audience” (Guan and Liu, 2019). The key distinction between audience and user lies in the fact that audiences passively receive information while users can actively make choices (Xia, 2017). The current Web 3.0 era represents an algorithmic age characterized by the widespread adoption and proliferation of algorithmic technologies (Kar and Moinuddin, 2025). Entering the algorithmic era, users’ initiative has not vanished. The existence of algorithmic awareness (Dogruel et al., 2020) enables them to retain control over the information flow (Swoot, 2021). Enhanced algorithmic awareness empowers users to choose how they interact with algorithmic systems (Hu and Ou, 2025). This indicates that users can still play an active role in shaping and controlling recommendation mechanisms to their advantage (Shin, 2021).

It can be seen from this that users in the Web 3.0 era remain active subjects who have the right to choose information, possess algorithmic awareness, and can interact with it. This indicates that the relationship between users and the information cocoon is not simply a matter of being “trapped.” To some extent, users should actively build and maintain their individual information cocoons.

Based on this, the limitations of existing research are primarily summarized as follows:

1. The logical foundation of existing research is rooted in Freud’s causal theory, which posits that an individual’s behavior is determined by some objective factor in the present or past (Freud and Satué, 1968). From this theoretical perspective, algorithmic stimuli and an individual’s past behavior are the causes of the information cocoons. This research perspective overlooks the user’s agency. Beyond external objective factors, users’ subjective factors also influence the formation of information cocoons.
2. The existing research focuses on external causes of information cocoons, attributing their emergence primarily to factors like information overload and algorithmic recommendations. However, the formation of information cocoons is not solely the result of external influences; internal factors such as users’ psychological needs and value judgments also play significant roles. Existing research lacks in-depth analysis of these internal factors, leaving studies confined to superficial external technologies and unable to explain the fundamental causes of information cocoons.
3. Within studies examining external causes, technological determinism has become the dominant explanatory paradigm. Many studies posit algorithms as the primary, if not sole, catalyst for information cocoons. This perspective isolates technology from users, overlooking how users in the Web3.0 era actively shape their information environment through behaviors like selection, blocking, and feedback.

Given existing research limitations, this study positions individuals as active agents and shifts focus to the intrinsic causes of information cocoons—specifically, the internal psychological motivations driving users to actively construct their own information cocoons.

### 2.3 Introduction of Adler’s teleology as a new perspective

This study introduces Adler’s teleology as a novel theoretical framework. Teleology posits that human actions are not predetermined by the past but guided by goals, with current behaviors serving merely as means to achieve objectives (Adler, 1928). This perspective fundamentally differs from “causalism” in its emphasis on subjective psychology. Freud attributed the emergence of behavior to objective factors, whereas Adler posited that behavior is determined by socially oriented subjective values or interests (Ansbacher and Ansbacher, 1964). That is, teleology holds that when confronted with external stimuli, individuals proactively select behavioral strategies based on their own goals. The three core concepts of Adler’s individual psychology provide a comprehensive theoretical framework for explaining users’ active construction of information cocoons:

First is the “creative self.” This concept posits that individuals do not passively respond to environmental influences but instead create their reality or behavioral patterns through subjective perception and interpretation of personal experiences (Sabates, 2020), developing distinct lifestyles based on their beliefs and goals (Watts, 2015). Applying this concept to information behavior implies that users do not passively receive algorithmic recommendations. Instead, driven by their creative self and guided by personal beliefs and goals, they actively filter information and shape their information environment.

Second is “social interest.” Adler posited that humans are social beings whose core need is to alleviate loneliness and gain a sense of belonging, connection, and integration through group bonds (Barlow et al., 2009). In the Web3.0 era, traditional offline socializing has shifted to online community interactions, with social networks becoming the primary vehicle for individuals to form connections based on shared interests and perspectives (Peniuk, 2023). Users’ behavior of constructing information cocoons fundamentally reflects the projection of social needs within the information environment. By filtering homogeneous information and integrating into like-minded online communities, they gain a sense of belonging and inclusion.

Finally, there is the intrinsic motivation of “striving for superiority.” Adler noted that individuals often harbor feelings of inferiority regarding their own capabilities. This inferiority does not stem from the information or situation itself, but from the individual’s subjective interpretation (Sabates, 2020). To compensate for this inferiority complex, individuals develop compensatory goals, which Adler defined as the “striving for superiority” (Adler, 1928). In summarizing Adler’s theory, Ansbacher states: “Behind all human activity lies a fundamental drive—the striving from a perceived state of deficiency toward fulfillment, from feelings of inferiority toward superiority, perfection, and wholeness” (Ansbacher and Ansbacher, 1964).

Applying this theory to information cocoons clarifies that they are not passive byproducts of human information behavior, but rather actively constructed outcomes driven by users’ creative selves, based on their psychological needs and goals.

### 2.4 Research framework construction and hypothesis formulation

#### 2.4.1 Six psychological motivations for users’ active construction of information cocoons

Based on Adler’s teleological concepts of “creative self,” “social interest,” and “striving for superiority,” this study identifies six psychological motivations for users’ active construction of information cocoons: sense of belonging, sense of control and security, maintenance of self-consistency, sense of superiority, goal orientation, and avoidance of failure and negative emotions.

##### 2.4.1.1 Sense of belonging

The theoretical foundation of belongingness is the concept of “social interest” in Adler’s teleology. This concept emphasizes the social nature of individuals and their connectedness to groups, indicating that individuals are not isolated entities but rather goal-oriented actors with the aim of “integrating into society.” Through participation in a particular system or environment, individuals can develop a sense of belonging, thereby perceiving themselves as an integral part of that system or environment (Pardede and Kovač, 2025; Hagerty et al., 1992). Essentially, all humans have a fundamental need for belonging that must be met by establishing and maintaining social connections (Pardede and Kovač, 2025). In the era of Web3.0, individuals’ social interest is projected onto cyberspace, making the need for belonging one of the motivations behind users’ information behavior. In information environments, users tend to isolate themselves within homogeneous and conceptually similar groups, preferring to interact with those who hold similar views (Cargnino and Neubaum, 2020). This behavioral strategy facilitates the formation of like-minded communities, allowing individuals to gain connection and recognition in virtual spaces (Del Vicario et al., 2016), thus satisfying their need for the sense of belonging.

##### 2.4.1.2 Sense of control and security

The concept of the “creative self” emphasizes an individual’s ability to actively shape their environment and behavioral patterns. The essence of this shaping lies in the “control” and “optimization” of one’s surroundings, aimed at fulfilling the internal need for a transition from uncertainty to certainty and from insecurity to security in personal life, thereby achieving a sense of overall control over life (Mereish et al., 2022). This sense of control is a significant driving force behind human behavior; people require predictable and controllable psychological states to avoid anxiety and chaos (Assari et al., 2025). In complex online environments, divergent information can increase users’ uncertainty and anxiety (Festinger, 1964). To maintain inner order and stability, users tend to selectively expose themselves to information that reinforces their existing beliefs, values, and preferences (Kar and Moinuddin, 2025), reducing cognitive dissonance and uncertainty, which in turn enhances their predictability and control over the information environment. This pursuit of control and security drives users to proactively construct a predictable and low-conflict information space to sustain psychological certainty and safety (Camaj, 2019).

##### 2.4.1.3 Self-consistency maintenance

The “creative self” prompts individuals to construct and maintain a unified and coherent lifestyle, the essence of which lies in the internal consistency between personality and cognition (Sabates, 2020; Adler, 1928). Adler used the metaphor of an “irregular triangle” to illustrate that regardless of its position, the fundamental nature of the triangle remains unified. However, complex and ever-changing external environments pose challenges to an individual’s internal stability. There should be some intrinsic motivation within the individual to preserve the unity and coherence of personality and cognition—that is, the self-consistency maintenance. In the era of big data characterized by information overload, human bounded rationality cannot cope with the vast amount of information on the internet, leading users to face issues of cognitive dissonance (Yuan and Wang, 2022). Such dissonance triggers a sense of discomfort (Hart et al., 2009), prompting users to avoid the psychological unease caused by cognitive conflict by disregarding information that significantly deviates from their own viewpoints (Gossart, 2014). Through this information filtering strategy, users are able to uphold internal unity amid complex informational environments, thereby achieving self-consistency maintenance.

##### 2.4.1.4 Superiority

Adler pointed out that feelings of inferiority are universal among individuals. To overcome this inferiority complex arising from perceived “imperfections” or “disadvantages,” individuals develop a strong internal drive known as the “striving for superiority.” They tend to engage in activities that allow them to feel superior to others (Imas and Madalas, 2024). This psychological tendency is widespread across different ages, genders, cultures, and dimensions of comparison (Hoorens et al., 2022). A sense of superiority is the external psychological manifestation of this striving; it is the self-validating and subjective experience that individuals gain through social comparison when they perceive themselves as advantaged in a particular domain (Zell et al., 2020). In the information environment, users focus their cognitive resources on specific areas to acquire and strengthen this sense of superiority. Information cocoons enable users to form cognitive advantages distinct from others within homogeneous content, thereby gaining recognition and ultimately compensating for their inferiority complex (Adler, 1928) to achieve the psychological goal of “striving for superiority.”

##### 2.4.1.5 Goal orientation

Teleology regards goals as central (Adler, 1928), with behavior merely serving as an adaptive means for individuals to employ different methods in pursuit of the same goal (Ansbacher and Ansbacher, 1964). Goal orientation is an internal tendency for individuals to proactively plan their actions based on clear objectives, representing an expression of intrinsic motivation (Van Dijk and Polani, 2013). This implies that human behavior is essentially goal-driven, and an individual’s expectations of future states guide their current choices and actions. Similarly, users require goal-oriented guidance within information environments. Bounded rationality theory posits that individuals’ limited cognitive capacities prevent them from processing all information with absolute rationality (Simon, 1947; Petracca, 2021). When confronted with massive amounts of information, users often search for and read only content relevant to their field while filtering out substantial amounts of information unrelated to their current goals, in order to locate useful information more quickly and efficiently (Yuan and Wang, 2022). By shaping an information environment aligned with their goals, users can rapidly advance tasks and achieve objectives within the constraints of bounded rationality. Goal orientation thus renders the information cocoon an effective tool for users to accomplish their purposes.

##### 2.4.1.6 Avoidance of failure and negative emotions

Avoidance of failure and negative emotions refers to a psychological tendency in which individuals engage in avoidance behaviors to prevent potential feelings of frustration, anxiety, or self-doubt (Wanchisen, 2018). Teleology suggests that an individual’s experience of inferiority can trigger an intrinsic drive toward “striving for superiority” (Adler, 1928). However, such negative emotions may also give rise to avoidance motives—when individuals encounter unfamiliar or opposing information, they may resort to avoidance behaviors to maintain their existing psychological equilibrium (Kardefelt-Winther, 2014). The virtual and selective nature of the internet facilitates this form of escape. When real-world pressures or negative emotions become overwhelming, users can turn to the internet as a substitute world where they do not feel threatened or challenged (Baturay and Tok, 2019). The formation of an information cocoon enables users to construct a cognitive comfort zone, allowing them to avoid psychological conflict and failure by blocking heterogeneous information and steering clear of unfamiliar domains.

Based on this, the paper proposes the first-level hypothesis regarding the main effects of the six psychological motivations:

*H1*: A sense of belonging positively influences users’ tendency to construct information cocoons.

*H2*: A sense of control and security positively influences users’ tendency to construct information cocoons.

*H3*: Self-consistency maintenance positively influences users’ tendency to construct information cocoons.

*H4*: Superiority positively influences users’ tendency to construct information cocoons.

*H5*: Goal orientation positively influences users’ tendency to construct information cocoons.

*H6*: Avoidance of failure and negative emotions positively influence users’ tendency to construct information cocoons.

#### 2.4.2 Two psychological motivational systems underlying users’ active construction of information cocoons

Based on Adler’s teleology and the intrinsic orientation of the six psychological dimensions, this study identifies two higher-level psychological motivational systems: the proactive motivational system and the defensive motivational system. This distinction stems from Adlerian theory’s interpretation of fundamental drives underlying human activity: On one hand, individuals possess an intrinsic drive to “pursue superiority.” This involves overcoming innate or acquired feelings of inferiority to achieve self-enhancement and self-worth. On the other hand, individuals possess the purpose of “seeking security and connection.” As social beings, individuals need to seek belonging through group connections, maintain self-consistency, and avoid external threats (Adler, 1928).

##### 2.4.2.1 Proactive motivation

This study categorizes superiority and goal orientation as proactive motivations. Both of these psychological motivations stem from the intrinsic drive to “pursue superiority,” which centers on individuals achieving self-improvement and accomplishments to compensate for feelings of inferiority and fulfill a positive desire for self-transcendence. Under this motivation, an information cocoon is not a closed cognitive barrier but an information environment actively shaped by users to achieve self-improvement. By filtering out irrelevant information, users can concentrate their limited cognitive resources on core goals or specific areas, ultimately achieving positive outcomes such as enhanced abilities and goal fulfillment.

##### 2.4.2.2 Defensive motivation

Defensive motivation encompasses four psychological dimensions: sense of belonging, control and security, maintaining self-consistency, and avoiding failure and negative emotions. These motivations stem from the individual’s desire to “seek safety and connection.” At their core, users integrate into groups sharing similar viewpoints and filter information aligned with their own cognition to avoid cognitive dissonance triggered by divergent content. This process maintains inner order and emotional stability, ultimately yielding a sense of belonging, security, and self-identity. Driven by these motivations, users construct information cocoons to cope with cognitive overload, simplifying complex information environments into familiar, controllable cognitive comfort zones. Information cocoons enable users to continuously receive homogeneous perspectives, reinforcing self-beliefs and reducing decision-making costs, while simultaneously avoiding the threat and discomfort associated with conflicting information.

Self-determination theory distinguishes between individuals’ intrinsic and extrinsic motivation, finding that enhancing extrinsic motivation can undermine an individual’s intrinsic motivation (Kuvaas et al., 2017). This study suggests that approach motivation and avoidance motivation are not independent of each other but instead jointly influence individuals’ information behaviors. That is, when a user’s approach motivation predominates, their information seeking may be more open, thereby weakening the influence of avoidance motivation; conversely, when avoidance motivation dominates, the user’s need for safety and stability may inhibit information retrieval driven by superiority or goal pursuit. Based on this, this study further proposes a second-level hypothesis regarding the moderating effect between the two major motivational systems:

*H7*: Proactive motivation moderates the influence of defensive motivation on users’ information cocooning behavior tendencies.

*H8*: Defensive motivation moderates the effect of proactive motivation on users’ information cocooning behavior tendencies.

#### 2.4.3 The moderating role of the six psychological motives

Self-determination theory (Ryan and Deci, 2000) posits that a balanced motivational system can only be achieved when three basic psychological needs—autonomy, competence, and relatedness—are dynamically satisfied. According to regulatory focus theory (Higgins, 1998), when defensive and approach motives coexist, individuals experience motivational competition due to limited cognitive resources, resulting in mutually inhibitory moderating effects. Therefore, this study further proposes a third-level hypothesis regarding the moderating effects of the six psychological motivation dimensions within the subsystem, examining the specific moderating mechanisms of each dimension at the system level:

*H9*: Goal orientation moderates the effect of defensive motivation on users’ tendency toward information cocooning behavior.

*H10*: Superiority moderates the effect of defensive motivation on users’ tendency toward information cocooning behavior.

*H11*: Sense of belonging moderates the effect of proactive motivation on users’ tendency toward information cocooning behavior.

*H12*: Sense of control and security jointly moderate the effect of proactive motivation on users’ tendency toward information cocooning behavior.

*H13*: Self-consistency maintenance moderates the effect of proactive motivation on users’ tendency toward information cocooning behavior.

*H14*: Avoidance of failure and negative emotions moderate the effect of proactive motivation on users’ tendency toward information cocooning behavior.

## 3 Research methodology

### 3.1 Participant inclusion and exclusion criteria

This study was conducted among Chinese university students, with the sample comprising undergraduates from freshman to senior years. Participants were recruited through two primary channels: offline distribution and collection of questionnaires at the author’s university, including locations such as academic buildings, cafeterias, and dormitories; and online sharing and referrals via social media platforms.

To ensure the samples are representative, specific inclusion and exclusion criteria were established. Participants must be currently enrolled undergraduate students from any year (freshman to senior) at a university in China, and they should have a basic understanding of the concept of the “information cocoon.” Before formally completing the survey, participants are required to complete a screening questionnaire on their comprehension of the information cocoon concept. This multi-dimensional screening tool consists of three modules: a knowledge test, scenario-based judgment, and a self-efficacy assessment. The knowledge test includes four multiple-choice questions, each worth 1 point: “What does the phenomenon of the information cocoon primarily refer to,” “What are the core characteristics of the information cocoon,” “How do algorithmic recommendations exacerbate the effect of the information cocoon,” “What are the main possible impacts of the information cocoon.”

The scenario-based judgment section contains three questions, each worth 1 point: “Xiao Ming enjoys watching technology-related videos, and the platform’s algorithm continuously recommends similar content, so he rarely encounters information from other fields. He finds this convenient, as it allows him to quickly access content of interest,” “Xiao Hong actively searches for news with different perspectives every day, including those that contradict her own views, to ensure she receives comprehensive information,” “Xiao Zhang only follows bloggers he agrees with, gradually becoming less exposed to differing opinions.”

The self-efficacy scale includes three items, each worth 1 point: “I can clearly explain the basic concept of the ‘information cocoon’,” “I can identify instances of the information cocoon phenomenon in social media,” “I understand the possible impacts of engaging in information cocoon behavior.” To ensure that participants possess a basic understanding of the information cocoon concept, they must correctly answer at least 3 out of 4 questions in the knowledge test, at least 2 out of 3 in the scenario-based judgment, and score≧2 out of 3 in the self-efficacy scale. Only those who meet all three criteria will pass the screening. To ensure that participants have a basic understanding of the concept of the information cocoon, they must correctly answer at least 3 questions on the knowledge test, at least 2 questions on the scenario judgment test, and score ≧12 on the self-assessment scale. Only those who meet all three criteria will pass the screening. In addition, to maintain consistency in information environment experiences, students who are enrolled solely in online or distance education programs, or who are studying abroad, were excluded from the analysis. Furthermore, individuals who were unfamiliar with or had little knowledge of the information cocoon were also excluded.

All participants were informed of the nature of the study and explicit informed consent must be obtained before participation in the study. They were given all necessary information regarding the ethical aspects of the research (e.g., anonymity, confidentiality, the right to withdraw from participation at any time, and the right to request data deletion after completion). Only undergraduate students aged 18 years or older who are native Chinese speakers were included in the study.

To ensure questionnaire quality, samples with excessively short (<2 min) or excessively long (>30 min) response times were removed.

The final sample comprised 807 participants, with males accounting for 48.6% and females for 51.4%.

The study revealed that regarding daily internet usage time: 69.1% of participants (*n* = 558) spent over 4 h daily online, 19% (*n* = 153) spent 3–4 h, 6.1% (*n* = 49) spent 2–3 h, 4.5% (*n* = 36) spent 1–2 h, and 1.4% (*n* = 11) spent 1 h or less. Additionally, regarding information acquisition via the internet, social platforms (rednote, douyin, WeChat, etc.) were reported as the most frequently used platforms for information gathering (97.8%, *n* = 789), followed by search engines (46.7%, *n* = 377). Other platforms (e.g., Bilibili) accounted for 4.2% of responses (*n* = 34).

### 3.2 Research design

This study primarily employed convenience sampling to collect questionnaire data. Data collection was conducted from September 1 to October 1, 2025, with a sample of 807 Chinese undergraduate students. All questionnaires were completed anonymously. Participants were drawn from various provinces across China, including Guangdong, Chongqing, Jiangsu, Zhejiang, Yunnan, Hunan, Shanxi, Fujian, Xinjiang, Anhui, Jiangxi, Sichuan, Jilin, Liaoning, and Shandong, with more than 20 universities covered offline. The questionnaire assessed participants’ demographic characteristics (gender, academic year) and information-seeking behaviors (average daily internet usage for information acquisition and primary platforms). Items were rated on a five-point Likert scale ranging from “strongly disagree/very weak” (1) to “strongly agree/very strong” (5).

The questionnaire measured “user information echo chamber tendencies” based on Sunstein’s (2006) Infotopia, comprising seven items: “I tend to focus on information within familiar domains,” “When encountering information that contradicts my existing views, I actively reduce exposure to such content,” “I frequently obtain information from a limited set of platforms,” “I am significantly more likely to click on platform recommendations aligned with my interests than on unfamiliar topics,” “Unless prompted by a specific task, I rarely proactively search for or browse information in unfamiliar domains,” “I adjust platform recommendation settings based on my preferences (e.g., blocking certain content, following specific accounts),” and “When friends or others share information unrelated to my interests, I typically do not read it in depth or seek further understanding.”

After measuring users’ behavioral tendencies toward information cocoons, the study developed separate items to assess the six primary psychological motivations driving users to construct information cocoons. The scale measuring sense of belonging referenced research by Cargnino and Neubaum (2020); items for sense of control and security drew from studies by Mereish et al. (2022), Kar and Moinuddin (2025), and others; Questions on self-consistency maintenance primarily referenced Festinger’s cognitive dissonance theory; questions on superiority referenced studies by Gibbons and Buunk (1999), Hoorens et al. (2022), Imas and Madalas (2024), and others; The goal orientation dimension draws from Van Dijk and Polani (2013) and Yuan and Wang (2022); avoidance of failure and negative emotions reference Kardefelt-Winther (2014) and Baturay and Tok (2019).

Respondents were instructed to answer based on personal experience. Additionally, expert input was sought to enhance questionnaire quality. The full questionnaire is provided in the Appendix of this study.

The data collection process adopted a single-blind design, meaning that participants were unaware of the specific hypotheses being tested. In addition, the data were anonymized, so analysts could not identify individual identities or access sensitive information through the data. The analysis process was not conducted under blind conditions.

### 3.3 Statistical analysis and data analysis strategy

Statistical analysis comprised the following: (i) Descriptive statistics for demographic variables (e.g., gender, grade level) alongside average daily internet information consumption duration, primary information platforms, and related variables (information cocooning tendencies, Adler-related dimensions), as shown in Table 1; (ii) Testing the reliability and validity of research variables, as presented in Tables 2, 3; (iii) Correlation analysis among primary variables, as shown in Table 4; (iv) Hypothesis testing employed a hierarchical analysis strategy: first, individual regression analyses (Table 5) verified the independent effects of each psychological motivation, followed by stepwise multiple linear regression analysis (Table 6) to identify core predictor variables within the full multivariate model. A forward selection method was applied, with an entry criterion of *p* < 0.05 and a removal criterion of *p* > 0.1. Motive dimensions were entered in descending order of explanatory power: sense of belonging, the sense of control and security, goal orientation, and avoidance of failure and negative emotions. (v) Proactive Motivation score = the mean of the sum of the superiority and goal orientation dimensions; Defensive motivation score = the mean of the sum of the four dimensions of sense of belonging, sense of control and security, self-consistency maintenance, and avoidance of failure and negative emotions. Both scores were standardized prior to analysis.

**Table 1:** Descriptive statistics of sample structure, average daily internet information consumption duration, primary information acquisition platforms, and related variables (*n* = 807).

Variable	
Gender (female, %)	415 (51.4)
Grade (Junior, %)	332 (41.1)
Daily internet usage time (mean, SD)	4.5 (0.90)
Number of internet platforms used (*n*, %)	
1 (social platform)	789 (97.8)
2 (news information)	274 (34.0)
3 (search engines)	377 (46.7)
4 (Internet forum)	240 (29.7)
5 (other)	34 (4.2)
Information cocoon behavior tendency (mean, SD)	3.77 (0.96)
Relevant dimensions (mean, SD)	
Sense of belonging	3.38 (1.04)
Sense of control and security	3.60 (0.96)
Self-consistency maintenance	3.42 (1.10)
Superiority	3.53 (1.07)
Goal orientation	3.78 (1.10)
Avoidance of failure and negative emotions	3.52 (1.04)

**Table 2:** Reliability and validity analysis.

Constructs	Items	Mean(SD)	Factor loadings	Cronbach’s alpha	KOM	CR	AVE
ICBT (information cocooning behavior tendency)	ICBT1	4.12 (0.992)	0.852	0.870	0.912	0.861	0.473
	ICBT2	3.33 (1.084)	0.548				
	ICBT3	4.25 (0.824)	0.874				
	ICBT4	4.14 (0.911)	0.862				
	ICBT5	3.54 (1.105)	0.598				
	ICBT6	3.57 (1.149)	0.599				
	ICBT7	3.39 (1.123)	0.539				
SOB (sense of belonging)	SOB1	3.30 (1.154)	0.637	0.866		0.866	0.520
	SOB2	3.83 (0.910)	0.791				
	SOB3	3.76 (0.834)	0.838				
	SOB4	3.72 (1.122)	0.641				
	SOB5	3.64 (0.914)	0.719				
	SOB6	3.78 (0.870)	0.789				
SOCAS (sense of control and security)	SOCAS1	3.52 (1.017)	0.565	0.816		0.814	0.469
	SOCAS2	3.57 (0.989)	0.752				
	SOCAS3	3.86 (0.997)	0.597				
	SOCAS4	3.79 (0.929)	0.747				
	SOCAS5	3.61 (0.884)	0.768				
SCM (self-consistency maintenance)	SCM1	3.74 (0.906)	0.787	0.832		0.836	0.505
	SCM2	3.60 (0.936)	0.714				
	SCM3	3.71 (0.965)	0.714				
	SCM4	3.78 (0.860)	0.805				
	SCM5	3.07 (1.238)	0.609				
S (superiority)	S1	3.82 (0.871)	0.803	0.855		0.853	0.537
	S2	3.62 (0.945)	0.688				
	S3	3.77 (0.834)	0.819				
	S4	3.53 (0.943)	0.736				
	S5	3.56 (1.018)	0.643				
GO (goal orientation)	GO1	3.98 (0.883)	0.808	0.881		0.881	0.597
	GO2	3.95 (0.881)	0.782				
	GO3	3.69 (0.898)	0.759				
	GO4	3.68 (0.920)	0.766				
	GO5	3.87 (0.842)	0.745				
AOFANE (avoidance of failure and negative emotions)	AOFANE1	3.33 (1.155)	0.777	0.919		0.922	0.631
	AFANE2	3.28 (1.064)	0.756				
	AFANE3	3.48 (1.013)	0.872				
	AFANE4	3.30 (1.106)	0.789				
	AFANE5	3.69 (0.971)	0.622				
	AFANE6	3.45 (1.015)	0.804				
	AFANE7	3.30 (1.070)	0.893				

SD, standard deviation; CR, composite reliability; AVE, average variance extracted.

**Table 3:** Fornell-Larker discrimination validity.

Constructs	ICBT	SOB	SOCAS	SCM	S	GO	AOFANE
ICBT	**0.688**						
SOB	0.725***	**0.721**					
SOCAS	0.671***	0.772***	**0.685**				
SCM	0.660***	0.805***	0.841***	**0.711**			
S	0.630***	0.764***	0.708***	0.807***	**0.733**		
GO	0.644***	0.695***	0.724***	0.735***	0.752***	**0.773**	
AOFANE	0.558***	0.699***	0.768***	0.786***	0.683***	0.643***	**0.794**

ICBT, information cocooning behavior tendency; SOB, sense of belonging; SOCAS, sense of control and security; SCM, self-consistency maintenance; S, superiority; GO, goal orientation; AOFANE, avoidance of failure and negative emotions; ***, **, * denote significance levels of 1, 5, and 10%, respectively; diagonal values represent the square root of the AVE. Bold values indicate the highest square root of AVE for each construct.

**Table 4:** Correlation matrix among key variables in this study (*n* = 807).

Constructs	1	2	3	4	5	6	7
1. ICBT	1	0.606**	0.592**	0.515**	0.489**	0.568**	0.361**
2. SOB		1	0.611**	0.577**	0.624**	0.617**	0.585**
3. SOCAS			1	0.660**	0.534**	0.570**	0.584**
4. SCM				1	0.563**	0.623**	0.573**
5. S					1	0.604**	0.490**
6. GO						1	0.521**
7. AOFANE							1
Gender	−0.052	−0.053	−0.044	−0.076*	−0.041	−0.117**	−0.008*
Grade	0.052	0.059	−0.051	0.102**	0.008	0.032	0.120**
Daily internet usage time	0.039	0.095**	−0.007	−0.044	0.052	−0.042	−0.038
Social platform	0.211**	0.120**	0.024	0.042	0.030	0.135**	−0.007
News information	−0.096**	0.149**	0.083*	0.125**	0.143**	0.192**	0.190**
Search engines	0.017	0.078*	−0.038	0.036	0.153**	0.130**	−0.018
Internet forum	−0.041	0.151**	0.055	0.035	0.035	0.008	0.042

Subdimensions of Adler’s teleology include sense of belonging (SOB), sense of control and security (SOCAS), self-consistency maintenance (SCM), superiority (S), goal orientation (GO), avoidance of failure and negative emotions (AOFANE); **Pearson zero-order correlation (*r*) significant at the 0.01 level (two-tailed test); *Pearson zero-order correlation (*r*) significant at the 0.05 level (two-tailed test); Gender reference = 1 (female).

**Table 5:** Independent effects analysis of six psychological motivations.

Information cocooning behavioral propensity	Coefficient (B)	Std. error	T-value	*p*-value	VIF
SOB	0.623***	0.029	21.445	0.000	1.016
SOCAS	0.620***	0.030	20.998	0.000	1.005
SCM	0.554***	0.033	16.956	0.000	1.017
S	0.509***	0.032	15.805	0.000	1.005
GO	0.578***	0.030	19.563	0.000	1.016
AOFANE	0.323***	0.030	10.860	0.000	1.021

SOB, sense of belonging; SOCAS, sense of control and security; SCM, self-consistency maintenance; S, superiority; GO, goal orientation; AOFANE, avoidance of failure and negative emotions; ***p < 0.001, **p < 0.01, *p < 0.05.

**Table 6:** Relationship between information cocooning behavior tendencies and key correlated predictors.

Predictors	Step 1				Step 2				Step 3				Step 4			
	B	SE	*β*	95%CI	B	SE	*β*	95%CI	B	SE	*β*	95%CI	B	SE	*β*	95%CI
SOB	0.623^***^	0.029	0.606^***^	[0.566, 0.679]	0.401^***^	0.034	0.391^***^	[0.335, 0.468]	0.303^***^	0.036	0.295^***^	[0.231, 0.374]	0.353^***^	0.037	0.343^***^	[0.279,0.426]
SOCAS					0.368^***^	0.035	0.353^***^	[0.300, 0.436]	0.296^***^	0.035	0.284^***^	[0.226, 0.365]	0.350^***^	0.037	0.336^***^	[0.278,0.422]
GO									0.227^***^	0.035	0.224^***^	[0.159, 0.295]	0.254^***^	0.035	0.251^***^	[0.187,0.322]
AOFANE													−0.149^***^	0.030	−0.166^***^	[−0.208,-0.090]
Model summary																
Variance explained by the model	R^2^ = 0.398 (39.8%)				R^2^ = 0.446 (44.6%)				R^2^ = 0.474 (47.4%)				R^2^ = 0.489 (48.9%)			
Change in variance by increasing step					∆R^2^ = 0.078 (7.8%)				∆R^2^ = 0.028 (2.8%)				∆R^2^ = 0.016 (1.6%)			
Statistical significance of the model	*F*_(1,805)_ = 468.383***				*F*_(2,804)_ = 323.318***				*F*_(3,803)_ = 241.137***				*F*_(4,802)_ = 192.233***			
Statistical significance of steps					ΔF_(1,804)_ = 113.055***				ΔF_(1,803)_ = 42.997***				ΔF_(1,802)_ = 24.422***			
VIF	1.971				2.948				3.924				4.884			

SOB, sense of belonging; SOCAS, sense of control and security; GO, goal orientation; AOFANE, avoidance of failure and negative emotions. ***p < 0.001; *p < 0.05, **p < 0.01; *p < 0.05. The following variables were excluded from the final model (i.e., Step 4) due to low predictive power or insignificance: gender (control variable), average daily time spent accessing information online, superiority, and self-consistency maintenance. B, unstandardized regression coefficient; SE, standard error; *β*, standardized regression coefficient; R^2^, coefficient of determination; ∆R^2^, change in coefficient of determination.

Before data collection, the required sample size was estimated using G*Power (version 3.1.9.7) to calculate the minimum sample size needed for the analysis (Faul et al., 2009). A medium effect size of f^2^ = 0.15, a statistical power of 1 − *β* = 0.8, and a significance level of *α* = 0.05 were preset. The analysis involved six predictor variables (i.e., sense of belonging, sense of control and security, self-consistency maintenance, superiority, goal orientation, and avoidance of failure and negative emotions) and two sociodemographic and usage-related variables (age, gender, and average daily duration of internet information acquisition). The results indicated that a sample size of 114 participants was required to achieve a power of 0.8.

#### 3.3.1 Reliability and validity

Since the model in this study involves measuring an abstract concept (latent variable) with multiple items, reliability was first assessed using composite reliability (CR) and Cronbach’ s alpha coefficient. Alpha. Hulland (1999) suggests that a composite reliability (CR) greater than 0.7 indicates internal consistency of the measured variable. In this study, the composite reliability of the latent variables ranged from 0.814 to 0.922 (see Table 2). Hair et al. (2017) propose that Cronbach’s Alpha above 0.7 indicates good construct reliability. Table 2 shows that all constructs’ Cronbach’s Alpha coefficients ranged from 0.816 to 0.919 (see Table 2), confirming the questionnaire’s strong reliability.

Next, this study examined convergent validity and discriminant validity. The research first assessed the scale’s KMO value to determine suitability for factor analysis. Results showed the construct’s KMO value of 0.912 (0.8 ≤ KMO ≤ 1) was appropriate for factor analysis. The study then conducted confirmatory factor analysis. Distinctness validity refers to the degree of correlation between different constructs; low correlations among constructs indicate clear differentiation, thus demonstrating distinctness validity. Aggregate validity aims to ensure all items under the same construct are highly correlated with that construct. This study employed partial least squares (PLS) to examine factor loadings and average variance extracted (AVE). Factor loadings ranged from 0.539 to 0.893 (see Table 2), indicating that these items possess convergent validity. However, as shown in Table 3, the square roots of the average variance extracted (AVE) for multiple constructs were smaller than the Pearson correlation coefficients for other constructs, indicating generally low discriminant validity between constructs. This result may suggest that these constructs measure a common, higher-order psychological structure, or that respondents do not psychologically distinguish these subtle dimensions clearly. Therefore, based on factor loadings and AVE values, weak items were removed from each item group. After revision, correlations between other constructs fell below the square root of each construct’ s AVE, indicating discriminant validity (see Table 7).

**Table 7:** Fornell-Larker discrimination validity (removing weak items from each item group).

Constructs	ICBT	SOB	SOCAS	COS	S	GO	AFANE
ICBT	0.879						
SOB	0.603***	0.708					
SOCAS	0.609***	0.657***	0.794				
SCM	0.568***	0.656***	0.763***	0.764			
S	0.533***	0.685***	0.659***	0.683***	0.747		
GO	0.612***	0.667***	0.645***	0.724***	0.727***	0.733	
AOFANE	0.366***	0.632***	0.673***	0.698***	0.611***	0.622***	0.816

ICBT, Information Cocooning Behavior Tendency; SOB, Sense of Belonging; SOCAS, Sense of Control and Security; SCM, self-consistency maintenance; S, Superiority; GO, Goal Orientation; AOFANE, Avoidance of failure and negative emotions. ***, **, and * denote significance levels of 1, 5, and 10%, respectively. Diagonal values represent the square root of the AVE.

To determine the applicability of the parametric approach, this study tested the assumptions of multiple linear regression analysis and used the variance inflation factor (VIF) to check for potential multicollinearity issues. All VIF values were less than or equal to 1.021, well below the threshold of 10, indicating no multicollinearity problems. The analysis was conducted using IBM SPSS Statistics version 26.

The moderation effects analysis was conducted using the PROCESS Procedure for SPSS Version 4.2 to explore the moderating effects of the two psychological motivation systems and six dimensions on users’ information cocooning behaviors.

## 4 Results

### 4.1 Common method bias test

A Harman’ s single-factor test was employed to assess common method bias (Zhou and Long, 2004). The results revealed nine factors with eigenvalues greater than 1, and the variance explained by the first factor was 38.021%, which is below the critical threshold of 40% (Xiong et al., 2012), indicating that there is no significant common method bias.

### 4.2 Descriptive statistics

Descriptive statistics, including means and standard deviations for all variables of interest, are presented in Table 1. Additionally, all sample characteristics are detailed in Table 1.

Results indicate that the study population primarily consists of third-year students (41.1%), with a nearly equal gender distribution among respondents (51.4, 48.6%). Respondents spent an average of over 4.5 h daily accessing information online. Primary platforms for information retrieval were social media (97.5%) and search engines (44.5%). Mean scores across all relevant dimensions exceeded 3 points. Thus, preliminary findings suggest that the six dimensions influence behavioral tendencies toward constructing information frameworks.

### 4.3 Correlation analysis

#### 4.3.1 Correlated factors and characteristics of ICBT

Pearson’s zero-order correlation analysis was conducted to examine the factors associated with ICBT (r), while accounting for all primary variables in this study (see Table 4). Overall, ICBT was positively correlated with a sense of belonging (*r* = 0.606, *p* < 0.01), a sense of control and security (*r* = 0.592, *p* < 0.01), self-consistency maintenance (*r* = 0.515, *p* < 0.01), superiority (*r* = 0.489, *p* < 0.01), goal orientation (*r* = 0.568, *p* < 0.01), and avoidance of failure and negative emotions (*r* = 0.361, *p* < 0.01). Correlation coefficients for respondents’ information acquisition via news platforms ranged from (*r* = 0.083, *p* < 0.01) (sense of control and security) to (*r* = 0.192, *p* < 0.01) (goal orientation). Daily internet usage duration for information acquisition (reference: 5 = 4 h and above) showed a correlation coefficient of (*r* = 0.095, *p* < 0.01) (sense of belonging).

### 4.4 Independent effects of six psychological motivations

This study adopted a hierarchical analysis strategy for hypothesis testing, first verifying the direct effects of the six psychological motivations (corresponding to H1-H6). Separate regression models were constructed for each of the six psychological motivations, with gender, grade level, and average time spent using the internet to obtain information included as control variables. Linear regression was used to test the six hypotheses. Results are presented in Table 5.

A sense of belonging (*B* = 0.623, *p* < 0.001) significantly and positively influences individuals’ behavioral tendency to construct information cocoons, meaning that as the sense of belonging increases, so does the behavioral tendency to build information cocoons. Hypothesis 1 is supported.

Sense of control and security (B = 0.620, *p* < 0.001) exerted a significant positive influence on individuals’ behavioral tendency to construct information cocoons. Specifically, as the sense of control and security increased, so did the behavioral tendency to construct information cocoons. Hypothesis 2 was supported.

Self-consistency maintenance (*B* = 0.554, *p* < 0.001) significantly and positively influences the tendency to construct information cocoons. Specifically, as the self-consistency maintenance increases, so does the tendency to construct information cocoons. Hypothesis 3 is supported.

Superiority (*B* = 0.509, *p* < 0.001) significantly and positively influences the tendency to construct information cocoons, meaning that stronger feelings of superiority enhance the tendency to actively build information cocoons. Hypothesis 4 is supported.

Goal orientation (*B* = 0.578, *p* < 0.001) significantly and positively influenced the tendency to construct information cocoons, meaning that stronger goal orientation led to a more pronounced tendency to actively construct information cocoons. Hypothesis 5 is supported.

Avoidance of failure and negative emotions (*B* = 0.323, *p* < 0.001) exerted a significant positive influence on the behavioral tendency to construct information cocoons. That is, the stronger the avoidance of failure and negative emotions, the more pronounced the behavioral tendency to actively construct information cocoons. Hypothesis 6 is supported.

### 4.5 Multiple linear regression

#### 4.5.1 Predictors of ICBT

To further evaluate the combined effects of all predictor variables, a complete regression model incorporating all six psychological motivations was established using stepwise multiple linear regression. The final model estimated in Step 4 (see Table 6) included sense of belonging (*β* = 0.343, *t* = 9.440), sense of control and security (*β* = 0.336, *t* = 9.557), goal orientation (*β* = 0.251, *t* = 7.357), avoidance of failure and negative emotions (*β* = −0.166, *t* = −4.942). All these variables were significant predictors of ICBT (*p* < 0.05), explaining approximately 48.9% of the total variance in ICBT scores, with a model VIF of 4.884 indicating no multicollinearity issues. Among all predictors, sense of belonging (*β* = 0.343) showed the strongest predictive contribution, with an incremental explanation of ∆R^2^ = 0.0016, ΔF[1,802] = 24.422, *p* = 0.000. The final model excluded two categories of variables: first, those with low predictive power or no statistical significance, including superiority (*p* = 0.225) and self-consistency maintenance (*p* = 0.124), indicating their effects may be masked by other psychological motivation dimensions. Second, control variables: gender (*p* > 0.05) and average daily internet information access duration (*p* = 0.603).

### 4.6 Moderating effects of two motivational systems

Based on the intrinsic orientation of the six psychological dimensions, the study further categorizes them into two higher-level motivational systems: the proactive motivational system and the reactive motivational system. Proactive motivation encompasses superiority and goal orientation, while reactive motivation includes a sense of belonging, a sense of control and security, maintenance of self-consistency, and avoidance of failure and negative emotions. Through moderation effect analysis, the interaction mechanism between systems (H7-H8) was verified.

To examine how proactive motivation moderates the strength of defensive motivation’s influence, we propose Hypothesis 7: Proactive motivation moderates the effect of defensive motivation on users’ tendency toward information silo behavior.

To examine how defensive motivation influences the strength of proactive motivation’s effect, we propose Hypothesis 8: Defensive motivation moderates the effect of proactive motivation on users’ information cocooning behavior tendencies.

Model 1 was significant: *F*_(3,803)_ = 271.5413, *p* = 0.000, R^2^ = 0.5036. Actual results indicate that the main effect of defensive motivation significantly and positively influences people’s tendency to actively construct information cocoons (*β* = 1.3650, *p* < 0.001). The interaction term between proactive motivation and defensive motivation significantly and negatively moderates this tendency (*β* = −0.2618, *p* < 0.001), indicating that proactive motivation negatively moderates the effect of defensive motivation on users’ information cocooning behavior—that is, proactive motivation weakens the influence of defensive motivation on such behavior. Hypothesis 7 is supported.

Model 2 was also significant: *F*_(3,803)_ = 271.5413, *p* = 0.000, R^2^ = 0.5036. Actual results indicate that the main effect of proactive motivation significantly positively influences the tendency to actively construct information cocoons (*β* = 1.2144, *p* < 0.000). The interaction term between defensive motivation and proactive motivation significantly negatively moderates this tendency (*β* = −0.2618, *p* < 0.001), indicating that defensive motivation negatively moderates the effect of proactive motivation on users’ information cocooning behavior—that is, defensive motivation weakens the influence of proactive motivation on such behavior. Hypothesis 8 is supported (Table 8).

**Table 8:** Analysis of the moderating effects of proactive and protective motivation.

ICBT	Model 1				Model 2			
	*β*	SE	*p*	95% CI	*β*	SE	*p*	95% CI
Defensive motivation	1.3650***	0.0874	0.0000	[1.1935, 1.5366]	1.3650***	0.0874	0.0000	[1.1935,1.5366]
Proactive motivation	1.2144***	0.0847	0.0000	[1.0480,1.3807]	1.2144***	0.0847	0.0000	[1.0480,1.3807]
Proactive motivation × Defensive motivation	−0.2618***	0.0218	0.0000	[−0.3045,-0.2191]	-			
Defensive motivation × Proactive motivation	-				−0.2618***	0.0027	0.0000	[−0.3045, −0.2191]
*F*	*F*_(3,803)_ = 271.5413***							
R-sq	R^2^ = 0.5036							

ICBT, information cocooning behavior tendency. ***p < 0.001, **p < 0.01, *p < 0.05.

### 4.7 Moderating effects of internal subdimensions in the two major motivational systems

Based on the negative moderating effects of the two major motivational systems, this study conducted separate moderation analyses for the six subdimensions to further explore the moderating relationships among core driving factors within each system.

#### 4.7.1 Moderating effects of goal orientation and superiority

To examine the relationship between the two dimensions of goal orientation and superiority and their influence on defensive motivation affecting users’ information cocooning behavior, Hypothesis 9 was proposed: Goal orientation moderates the effect of defensive motivation on users’ tendency toward information cocooning behavior; Hypothesis 10: Superiority moderates the effect of defensive motivation on users’ tendency toward information cocooning behavior.

Model 3 was significant: *F*_(3,803)_ = 268.0948, *p* = 0.000, R^2^ = 0.5004. Actual results indicate that the main effect of defensive motivation significantly positively influences people’s tendency to actively construct information cocoons (*β* = 1.3286, *p* < 0.001). The interaction term GO x defensive motivation significantly negatively moderates this tendency (*β* = −0.2374, *p* < 0.001), indicating that goal orientation negatively moderates the effect of defensive motivation on information cocooning behavior. Specifically, stronger goal orientation weakens the promotional effect of defensive motivation on users’ information cocooning behavior. Hypothesis 9 is supported.

Model 4 was significant: *F*_(3,803)_ = 245.9560, *p* = 0.000, R^2^ = 0.4789. Actual results show that the main effect of defensive motivation significantly positively influences the tendency to actively construct information cocoons (*β* = 1.5094, *p* < 0.001). The interaction term S x defensive motivation significantly negatively moderates this tendency (*β* = −0.2704, *p* < 0.001), indicating that superiority negatively moderates the effect of defensive motivation on information cocooning behavior. That is, the pursuit of superiority inhibits the behavioral tendency driven by defensive motivation when users construct information cocoons. Hypothesis 10 is supported (Table 9).

**Table 9:** Analysis of moderating effects of goal orientation and superiority.

ICBT	Model 3				Model 4			
	*β*	SE	*p*	95% CI	*β*	SE	*p*	95% CI
Defensive motivation	1.3286***	0.0815	0.0000	[1.1686, 1.4886]	1.5094***	0.0860	0.0000	[1.3406, 1.6782]
GO	1.0497***	0.0748	0.0000	[0.9029, 1.1965]	-			
S	-				1.1207***	0.0893	0.0000	[0.9453,1.2960]
GO×Defensive Motivation	−0.2374***	0.0230	0.0000	[−0.2772,-0.1975]	-			
S × Defensive Motivation	-				−0.2704***	0.0230	0.0000	[−0.3155,-0.2252]
*F*	*F*_(3,803)_ = 268.0948***				*F*_(3,803)_ = 245.9560***			
R-sq	R^2^ = 0.5004				R^2^ = 0.4789			

ICBT, information cocooning behavior tendency; S, superiority; GO, goal orientation. ***p < 0.001, **p < 0.01, *p < 0.05.

#### 4.7.2 Moderating effects of belonging, control and security

To investigate the relationship between the two dimensions of sense of belonging, sense of control and security, and their impact on approach motivation in relation to users’ information cocoon behavior, we propose Hypothesis 11: Sense of belonging moderates the effect of proactive motivation on users’ tendency toward information cocooning behavior; Hypothesis 12: Sense of control and security jointly moderate the effect of proactive motivation on users’ tendency toward information cocooning behavior.

Model 5 was significant: *F*_(3,803)_ = 266.8071, *p* = 0.000, R^2^ = 0.4992. Actual results indicate that the main effect of proactive motivation significantly and positively influences people’s tendency to actively construct information cocoons (*β* = 1.1337, *p* < 0.001). The interaction term SOB x proactive motivation exerts a significant negative moderating effect on this tendency (*β* = −0.2264, *p* < 0.001), indicating that sense of belonging negatively moderates the effect of proactive motivation on information cocooning behavior. Specifically, higher levels of sense of belonging weaken the promotional effect of proactive motivation on users’ tendency to actively construct information cocoons. Hypothesis 11 is supported.

Model 6 was significant: *F*_(3,803)_ = 303.1959, *p* = 0.000, R^2^ = 0.5311. Actual results indicate that the main effect of proactive motivation significantly and positively influences the tendency to actively construct information cocoons (*β* = 1.2605, *p* < 0.001). The interaction term SOCAS x proactive motivation exerts a significant negative moderating effect on this tendency (*β* = −0.2563, *p* < 0.001), indicating that perceived control and security negatively moderate the influence of proactive motivation on information cocooning behavior. Specifically, stronger perceived control and security weaken the psychological motivational impact of proactive motivation on users’ information cocooning behavior. Hypothesis 12 is supported (Table 10).

**Table 10:** Analysis of the moderating effects of sense of belonging, sense of control and security.

ICBT	Model 5				Model 6			
	*β*	SE	*p*	95% CI	*β*	SE	*p*	95% CI
Proactive motivation	1.1337***	0.0795	0.0000	[0.9776, 1.2897]	1.2605***	0.0737	0.0000	[1.1158,1.4053]
SOB	1.1373***	0.0768	0.0000	[0.9866,1.2880]	-			
SOCAS	-				1.2793***	0.0760	0.0000	[1.1300,1.4286]
SOB × Proactive motivation	−0.2264***	0.0206	0.0000	[−0.2668, −0.1859]	-			
SOCAS × Proactive motivation	-				−0.2563***	0.0197	0.0000	[−0.2950, −0.2176]
*F*	*F*_(3,803)_ = 266.8071***				*F*_(3,803)_ = 303.1959***			
R-sq	R^2^ = 0.4992				R^2^ = 0.5311			

ICBT, information cocooning behavior tendency; SOB, sense of belonging; SOCAS, sense of control and security. ***p < 0.001, **p < 0.01, *p < 0.05.

#### 4.7.3 Moderating effects of self-consistency maintenance, avoidance of failure and negative emotions

To investigate the relationship between self-consistency maintenance, failure avoidance, negative emotions, and their respective effects on the influence of proactive motivation on users’ information cocooning behavior, we propose Hypothesis 13: Self-consistency maintenance moderates the effect of proactive motivation on users’ tendency toward information cocooning behavior; Hypothesis 14: Avoidance of failure and negative emotions moderate the effect of proactive motivation on users’ tendency toward information cocooning behavior.

Model 7 was significant: *F*_(3,803)_ = 217.8022, *p* = 0.000, R^2^ = 0.4486. Actual results indicate that the main effect of proactive motivation significantly positively influences people’s tendency to actively construct information cocoons (*β* = 1.2907, *p* < 0.001). The interaction term SCM x proactive motivation significantly negatively moderates users’ tendency to actively construct information cocoons (*β* = −0.2233, *p* < 0.001), indicating that self-consistency maintenance negatively moderates the effect of proactive motivation on users’ information cocooning behavior. Specifically, stronger self-consistency maintenance weakens the influence of proactive motivation on users’ active information cocooning. Hypothesis 13 is supported.

Model 8 was significant: *F*_(3,803)_ = 198.7809, *p* = 0.000, R^2^ = 0. 4,262. Actual results indicate that the main effect of proactive motivation significantly positively influences the tendency to actively construct information cocoons (*β* = 1.3591, *p* < 0.001). The interaction term AOFANE x proactive motivation exerts a significant negative moderating effect on this tendency (*β* = −0.2352, *p* < 0.001), indicating that avoidance of failure and negative emotions negatively moderate the effect of proactive motivation on users’ information cocooning behavior. That is, the more users tend to avoid failure and negative emotions, the weaker the influence of proactive motivation on their information cocooning behavior. Hypothesis 14 is supported (Table 11).

**Table 11:** Analysis of the moderating effects of self-consistency maintenance and avoidance of failure and negative emotions.

ICBT	Model 7				Model 8			
	*β*	SE	*p*	95% CI	*β*	SE	*p*	95% CI
Proactive motivation	1.2907***	0.0858	0.0000	[1.1223, 1.4590]	1.3591***	0.0780	0.0000	[1.2060,1.5122]
SCM	0.9852***	0.0820	0.0000	[0.8242,1.1462]	-			
AOFANE	-				0.9449***	0.0928	0.0000	[0.7628,1.1270]
SCM × Proactive motivation	−0.2233***	0.0217	0.0000	[−0.2659, −0.1806]	-			
AOFANE × Proactive motivation	-				−0.2352***	0.0228	0.0000	[−0.2800, −0.1905]
*F*	*F*_(3,803)_ = 217.8022***				*F*_(3,803)_ = 198.7809 ***			
R-sq	R^2^ = 0.4486				R^2^ = 0.4262			

ICBT, information cocooning behavior tendency; SCM, self-consistency maintenance; AOFANE, avoidance of failure and negative emotions. ***p < 0.001, **p < 0.01, *p < 0.05.

## 5 Discussion

### 5.1 Research findings

This study aims to explore the psychological motivations underlying users’ tendency to actively construct information cocoons, and to examine both the independent effects of the six psychological motives on information cocoon behavior and their moderating interactions within the system. Through data analysis, the following conclusions were drawn.

First, differences between single-variable and stepwise regression results reveal the complexity of psychological motivation systems. Linear regression analyses of the six major psychological motivations individually showed that all significantly and positively influenced users’ tendency to actively construct information cocoons. This indicates that users’ online information selection is not entirely random or passive; it is directly driven by their intrinsic psychological motivations. This confirms that users do exhibit a tendency to actively construct information cocoons, supported by multiple psychological motivations.

Furthermore, although “superiority” and “self-consistency maintenance” both significantly influenced users’ active construction of information cocoons in individual tests, their explanatory power significantly weakened when all six psychological motivations were incorporated into a comprehensive regression model. This suggests that the psychological motivation system is complex and dynamic. Different motivations may interact synergistically to produce information cocoons when their goals for information filtering converge. Alternatively, when users allocate limited attention to a single behavior, the intensification of one motivation may weaken the effects of another through mutual inhibition. These factors cause the independent effects of certain motivations to diminish in scenarios where multiple motivations coexist.

The study validated the foundational driving role of six psychological motivations in users’ proactive information cocooning behavior through separate tests and stepwise multiple linear regression models, revealing the dynamic characteristics of effects within a multi-motivation system. For instance, both “superiority” and “maintaining self-consistency” functioned as drivers in individual tests. However, when coexisting, users may reduce reliance on either motive to integrate the conflicting behavioral goals of “superiority” and “consistency,” thereby weakening their respective effects. Consequently, the study focused on the moderating relationships among psychological motives to further explore their interactive mechanisms. Through moderation effect analysis, the study found that proactive motivations (superiority and goal orientation) and defensive motivations (sense of belonging, sense of control and security, self-consistency maintenance, avoidance of failure and negative emotions) exert mutually inhibitory moderating effects on information cocooning behavior. Specifically, when users exhibit strong proactive motivation, it weakens the facilitative effect of defensive motivation on information cocooning behavior. Conversely, when defensive motivation is strong, it similarly inhibits the information exploration tendencies driven by proactive motivation. This indicates that when both proactive and defensive motivations simultaneously influence users’ information cocooning behavior, stronger proactive motivation enables users to fully utilize relevant information and actively step outside their comfort zones. Conversely, when defensive motivation dominates, users tend to remain within their comfortable and secure information circles. This finding aligns with Higgins’ (1998) research on the “Regulatory Focus Theory.” This perspective posits that individuals with a promotion focus actively pursue goal attainment and acquisition, while those with a prevention focus avoid and mitigate related mismatches. This implies that when confronted with diverse online information, users may activate a promotion focus—generating proactive motivation—driven by pursuits of superiority or goal attainment. Alternatively, they may activate a prevention focus—generating defensive motivation—driven by needs for belonging, control, security, self-consistency maintenance, or avoidance of failure and negative emotions. This state emerges under specific contextual conditions and individual user traits. Further integrating the moderated focus theory, this “mutual inhibition” fundamentally reflects the motivational conflict and resource competition between promotion and prevention focuses. Given that an individual’s cognitive resources and attention span are finite, when proactive motivation (promotion focus) is highly activated, psychological energy is prioritized toward goals of “seeking gains or superiority” and “growth and progress.” Consequently, the psychological resources allocated to sustaining reactive motivation (prevention focus) diminish, thereby weakening the driving force of reactive motivation behind information avoidance behavior. Conversely, when the defensive focus dominates, individuals enter a “risk-vigilant” psychological state. This state automatically filters out external stimuli that may trigger uncertainty (i.e., exploring new information), thereby inhibiting the risk-taking and exploratory behaviors advocated by the proactive motivation. This flexible motivational switching mechanism results in users’ final information behaviors being the outcome of a dynamic equilibrium achieved through mutual inhibition between these two motivational forces.

Furthermore, research reveals that all six psychological dimensions negatively moderate the influence of their corresponding psychological motivation systems on users’ proactive information cocooning behaviors. This indicates these sub-psychological motivations alter the intensity or direction of the relationship between psychological motivation systems and users’ information cocooning behaviors. For instance, when levels of control and security are high, the effect of proactive motivation on users’ information cocooning behaviors is weakened. This indicates that psychological dimension factors are key conditions influencing the conversion of psychological motivation into behavior.

The study further found that the moderation strength of each dimension within defensive motivation is roughly equivalent to that of each dimension within proactive motivation, indicating no inherent superiority of one psychological motivation type over the other. Whether defensive or proactive motivation, the moderation strength of their associated psychological dimensions on users’ information cocooning behavior shows no significant difference. This demonstrates that both major motivation systems exert equally important influences on user behavior. Dual Motivation Theory posits that intrinsic and extrinsic motivations are not mutually exclusive but exist on a continuum, involving joint regulation from both external and internal factors. This supports our study’s conclusion: defensive and proactive motivations hold no hierarchical priority in users’ active information cocooning behaviors. They jointly shape such behaviors through distinct psychological dimensions with comparable moderating strengths.

Notably, while superiority (a proactive motive) and self-consistency maintenance (a defensive motive) showed significant effects in individual tests, their explanatory power weakened in multivariable contexts. This finding further supports the conclusion that the moderating strength of defensive motives balances that of proactive motives. Consistent with dual-motivation theory, when users face multiple psychological needs simultaneously, both the defensive motivation system and the proactive motivation system may play equally fundamental and central roles in users’ information cocooning behavior under the influence of different psychological dimensions.

### 5.2 Theoretical implications

This study incorporates Adler’s teleology, conceptualizing information cocoons as proactive behavioral strategies users adopt to achieve specific psychological goals. It identifies six psychological dimensions underpinning users’ active construction of information cocoons, categorizing these dimensions into two motivational systems: proactive and defensive. Analysis of the moderating effects of these two motivational systems on the six psychological dimensions reveals that users’ proactive information cocooning may stem from either defensive or proactive motivations. This finding demonstrates that the endogenous nature of psychological motivations influences information cocooning behavior through complex interrelated mechanisms, offering a novel research pathway for understanding the causes of information cocooning.

### 5.3 Practical implications

First, users can be guided to recognize their psychological motivations during information selection. By leveraging the mutual constraints between the two motivational systems, users can actively “break out of their information cocoons.” Second, relevant authorities should focus on users’ psychological motivations alongside algorithmic recommendations when conducting online regulation, rather than attributing issues solely to algorithmic factors. When users’ defensive motivation outweighs their exploratory motivation, efforts should focus on emotional regulation and cognitive restructuring to enhance psychological resilience, helping users develop more positive and adaptive thinking patterns. Conversely, when exploratory motivation dominates, users should be guided to elevate their “pursuit of excellence” from “achieving the best within existing cognitive boundaries” to “expanding beyond cognitive limits toward more comprehensive development.”

### 5.4 Research limitations

Despite its contributions, this study has certain limitations. First, it did not control for potential confounding variables such as geographical location, urban–rural differences, and socioeconomic background among research subjects, nor did it report sample attrition rates. These factors may introduce bias and affect the generalizability of results. Second, the six-dimensional factors used in this study do not encompass all psychological motivations. Therefore, future research could explore whether other psychological motivations beyond these six dimensions significantly influence users’ information cocooning behaviors. Second, data collection and surveys were conducted at a specific point in time, making it impossible to determine whether long-term outcomes would change over time. Third, although the CR values of the scales were reliable, their discriminant validity was insufficient. Future research will focus on further refining and clarifying the measurement items for these constructs. Fourth, as shown in Table 4, the correlation strength among the six motives is relatively high, suggesting that they may be driven by common underlying needs and are likely to coexist. Based on the linear models established in this study, the fundamental relationships among the variables were identified. Future research could build upon the preliminary path hypotheses derived from the hierarchical regression results to construct a structural equation model with defensive motivation and approach motivation as latent variables, thereby further exploring the mediating mechanisms and more complex network relationships among the motives. Finally, this study’s respondents comprised undergraduate students from freshman to senior years, representing a relatively homogeneous age cohort. However, information-seeking habits vary across age groups, and user behavioral tendencies may differ based on demographic characteristics, personal experiences, and information-access patterns. Consequently, future research should target diverse age cohorts and extend this model to incorporate additional variables and diverse cultural or national contexts.

## Data Availability

The original contributions presented in the study are included in the article/Supplementary material, further inquiries can be directed to the corresponding author.
