# Supplementary material for: The psychological motivation of users actively constructing information cocoons from the perspective of Adler’s teleology: an empirical study based on a sample of Chinese university students
Source: Front Psychol. 2026 Apr 24;17:1742055. doi: 10.3389/fpsyg.2026.1742055 (PMC13159072; doi:10.3389/fpsyg.2026.1742055)
Supplement: Supplementary file 1 [file Supplementary_File_1.zip › ▓╣│Σ▓─┴╧appendix/Survey Questionnaire Chinese version.docx]

《从阿德勒目的论视角探究用户主动构建信息茧房的动机》调查问卷

尊敬的受访者：​

您好！本问卷旨在探究用户主动构建信息茧房的动机。信息茧房指个体在信息选择中，基于个人兴趣、价值观或算法推荐，主动或被动地排斥异质信息，长期沉浸于同质化内容中，形成如“蚕茧”般的封闭信息环境，导致认知局限、观点极化与社会共识割裂。本问卷实行匿名填写，所有数据仅用于学术研究，问卷填写约需8-10分钟，请您根据实际情况如实作答。感谢您的支持与配合！

一、基本信息

1.您的性别：​

□ 男 □ 女

2.您的年级：​

□ 大一 □ 大二 □ 大三 □ 大四

3.您平均每天使用互联网获取信息的时长：​

□ 1 小时及以下 □ 1-2 小时 □ 2-3 小时 □ 3-4 小时 □ 4 小时以上​

4.您主要通过哪些平台获取信息（可多选）：​

□ 社交平台（如微信、小红书、抖音等） □ 新闻资讯平台（如今日头条、腾讯新闻等） □ 搜索引擎（如百度、谷歌等） □ 专业论坛 / 社群（如知乎、豆瓣小组等）

1. 信息茧房行为倾向
2. 我倾向于关注自己熟悉领域的信息（ ）

A.完全不符合 B.不太符合 C.一般 D.比较符合 E.完全符合

1. 当遇到与自己现有观点相悖的信息时，我会主动减少对这类信息的关注（ ）

A.完全不符合 B.不太符合 C.一般 D.比较符合 E.完全符合

3.我经常在固定的几个平台上获取信息（ ）

A.完全不符合 B.不太符合 C.一般 D.比较符合 E.完全符合

4.若平台推荐的信息与我的兴趣匹配，我点击查看的概率会显著高于陌生主题信息（ ）
A.完全不符合 B.不太符合 C.一般 D.比较符合 E.完全符合

5.除非有明确任务要求，否则我很少主动搜索或浏览陌生领域的信息（ ）

A.完全不符合 B.不太符合 C.一般 D.比较符合 E.完全符合

6.我会根据自己的信息偏好，调整平台的推荐设置（如屏蔽某类内容、关注特定账号）（ ）

A.完全不符合 B.不太符合 C.一般 D.比较符合 E.完全符合

7.当朋友或他人分享与我兴趣无关的信息时，我通常不会深入阅读或进一步了解（）

A.完全不符合 B.不太符合 C.一般 D.比较符合 E.完全符合

1. 阿德勒目的论相关维度
2. 归属感
3. 我长期活跃于某个或某几个固定的网络社群（如粉丝群、论坛版块、游戏公会等），并对其有强烈的归属感（ ）

A.完全不符合 B.不太符合 C.一般 D.比较符合 E.完全符合

2.当我持有的观点在所属网络社群中得到广泛赞同时，我会感到非常安心和满足（ ）

A.完全不符合 B.不太符合 C.一般 D.比较符合 E.完全符合

3.当我获取到与自己兴趣一致的信息时，会产生 “我属于这个信息圈子” 的归属感（ ）

A.完全不符合 B.不太符合 C.一般 D.比较符合 E.完全符合

4.我会为了避免与我所在网络社群的主流观点冲突，而选择不发表或少发表不同意见（ ）

A.完全不符合 B.不太符合 C.一般 D.比较符合 E.完全符合

5.我会主动关注当下大家普遍讨论的热门信息类型，以获得社交中的共同话题（ ）

A.完全不符合 B.不太符合 C.一般 D.比较符合 E.完全符合

6.若某类信息能让我与身边重要的人保持话题一致，我会更愿意主动获取这类信息（ ）

A.完全不符合 B.不太符合 C.一般 D.比较符合 E.完全符合

（二）掌控感和安全感

1.面对互联网上海量且常常相互矛盾的信息，我时常感到困惑和焦虑（ ）

A.完全不符合 B.不太符合 C.一般 D.比较符合 E.完全符合

2.我更喜欢关注那些结论明确、符合我预期的话题和内容，这让我觉得一切尽在掌握（ ）

A.完全不符合 B.不太符合 C.一般 D.比较符合 E.完全符合

3.我有意识地取关或屏蔽那些经常发布让我不舒服或难以认同的信息的账号（ ）

A.完全不符合 B.不太符合 C.一般 D.比较符合 E.完全符合

4.我倾向于信任少数几个我认为“可靠”的信息源，这比我自己从混乱信息中判断要省心得多（ ）

A.完全不符合 B.不太符合 C.一般 D.比较符合 E.完全符合

5.当我习惯使用的信息平台或账号突然改变风格或推送我不感兴趣的内容时，我会感到不适（ ）

A.完全不符合 B.不太符合 C.一般 D.比较符合 E.完全符合

（三）自我一致性的维持

1.我相信的信息，通常是那些能够证实和强化我原有想法和价值观的信息（ ）

A.完全不符合 B.不太符合 C.一般 D.比较符合 E.完全符合

2.如果看到与我核心信念相左的有力证据，我的第一反应是去质疑证据的来源或可靠性，而非质疑自身信念（ ）

A.完全不符合 B.不太符合 C.一般 D.比较符合 E.完全符合

3.接触太多反面信息，会让我怀疑自己的判断力，这种感觉很不好（ ）

A.完全不符合 B.不太符合 C.一般 D.比较符合 E.完全符合

4.当我认同的观点被当作“真理”广泛传播时，我会感到自己的价值和智慧得到了肯定（ ）

A.完全不符合 B.不太符合 C.一般 D.比较符合 E.完全符合

1. 我会倾向于将复杂的道德或社会问题简单化为“善与恶”、“对与错”的二元对立，因为这更符合我已有的认知框架（ ）

A.完全不符合 B.不太符合 C.一般 D.比较符合 E.完全符合

1. 优越感
2. 我希望通过深入积累某一领域的信息，让自己在该领域的知识储备超过身边大多数人（ ）

A.完全不符合 B.不太符合 C.一般 D.比较符合 E.完全符合

2.当我在特定信息领域（如游戏、美妆）掌握更多细节知识时，会产生“比他人更懂这方面” 的优越感（ ）

A.完全不符合 B.不太符合 C.一般 D.比较符合 E.完全符合

3.我倾向于在自己擅长的信息领域持续深耕，通过分享该领域信息凸显自己的独特价值（ ）

A.完全不符合 B.不太符合 C.一般 D.比较符合 E.完全符合

4.我关注的信息源让我感觉“我一直在学习/进步”，即使这些信息同质化很高（ ）

A.完全不符合 B.不太符合 C.一般 D.比较符合 E.完全符合

5.我会刻意关注某类有门槛的信息（如专业术语、小众文化），以区别于“普通信息消费者”（ ）

A.完全不符合 B.不太符合 C.一般 D.比较符合 E.完全符合

1. 目标导向

1.我主动关注特定类型信息（如考研资料、职场技能），是为了实现学习、工作或生活中的明确目标（ ）

A.完全不符合 B.不太符合 C.一般 D.比较符合 E.完全符合

2.在开始获取信息前，我会先明确自己的目标（如了解某事件真相、学习某项技能），再筛选对应信息类型（ ）

A.完全不符合 B.不太符合 C.一般 D.比较符合 E.完全符合

3.为了高效达成目标，我会集中精力获取与目标直接相关的信息，忽略无关内容（ ）

A.完全不符合 B.不太符合 C.一般 D.比较符合 E.完全符合

4.若某类信息无法为我的目标提供支持（如学习目标下的娱乐信息），我会主动减少对这类信息的投入时间（ ）

A.完全不符合 B.不太符合 C.一般 D.比较符合 E.完全符合

5.我会根据目标的优先级调整信息获取方向（ ）

A.完全不符合 B.不太符合 C.一般 D.比较符合 E.完全符合

1. 逃避失败与负面情绪
2. 我避开陌生领域信息（如量子物理、金融知识），是担心自己无法理解这些内容而产生“学不会”的挫败感（ ）

A.完全不符合 B.不太符合 C.一般 D.比较符合 E.完全符合

2.面对与自己观点不同的信息时，我会主动回避（ ）

A.完全不符合 B.不太符合 C.一般 D.比较符合 E.完全符合

3.我更愿意关注自己熟悉的信息，是因为接触新信息时会产生“不确定能否掌握”的不安感（ ）

A.完全不符合 B.不太符合 C.一般 D.比较符合 E.完全符合

4.当接触到复杂且陌生的信息时，我会因害怕无法完全理解而选择放弃深入了解（ ）

A.完全不符合 B.不太符合 C.一般 D.比较符合 E.完全符合

5.若某类信息（如负面新闻、争议话题）可能引发我的烦躁或焦虑情绪，我会主动屏蔽这类信息（ ）

A.完全不符合 B.不太符合 C.一般 D.比较符合 E.完全符合

6.我主动将信息获取范围固定在有限领域，是为了避免面对海量信息时“不知道该选什么”的焦虑感（ ）

A.完全不符合 B.不太符合 C.一般 D.比较符合 E.完全符合

7.我避免接触与自己现有认知冲突的信息，是担心这些信息会打破我的认知习惯，导致“自我怀疑”的负面感受（ ）

A.完全不符合 B.不太符合 C.一般 D.比较符合 E.完全符合

再次感谢您抽出宝贵时间完成问卷！您的回答对本研究的科学性至关重要，祝您生活愉快！​
