# Supplementary material for: The psychological motivation of users actively constructing information cocoons from the perspective of Adler’s teleology: an empirical study based on a sample of Chinese university students
Source: Front Psychol. 2026 Apr 24;17:1742055. doi: 10.3389/fpsyg.2026.1742055 (PMC13159072; doi:10.3389/fpsyg.2026.1742055)
Supplement: Supplementary file 1 [file Supplementary_File_1.zip › ▓╣│Σ▓─┴╧appendix/Survey Questionnaire English version.docx]

Appendix

Survey Questionnaire

Dear Respondent:

Hello! This questionnaire aims to explore the motivations behind users actively constructing information cocoons. An information silo refers to an individual’s deliberate or passive exclusion of diverse information based on personal interests, values, or algorithmic recommendations, leading to prolonged immersion in homogeneous content. This creates a closed information environment resembling a “silk cocoon,” resulting in cognitive limitations, viewpoint polarization, and fragmentation of social consensus. This questionnaire is anonymous. All data will be used solely for academic research. Completion takes approximately 8-10 minutes. Please answer truthfully based on your actual circumstances. Thank you for your support and cooperation!

I. Basic Information

1. Your Gender:

□ Male □ Female

2. Your academic year:​

□ Freshman □ Sophomore □ Junior □ Senior

3. Average daily time spent accessing information online:​

□ 1 hour or less □ 1-2 hours □ 2-3 hours □ 3-4 hours □ Over 4 hours

4. Which platforms do you primarily use to access information? (multiple selections allowed):​

□ Social media platforms (e.g., WeChat, Xiaohongshu, Douyin) □ News and information platforms (e.g., Toutiao, Tencent News)

□ Search engines (e.g., Baidu, Google) □ Professional forums/communities (e.g., Zhihu, Douban Groups)

II. Information Echo Chamber Behavior Tendencies

1. I tend to focus on information within my familiar fields ( )

A. Not at all applicable B. Not very applicable C. Somewhat applicable D. Fairly applicable E. Completely applicable

2. When encountering information that contradicts my existing views, I actively reduce my exposure to such content ( )

A. Completely disagree B. Somewhat disagree C. Neutral D. Somewhat agree E. Completely agree

3. I frequently obtain information from a fixed set of platforms ( )

A. Completely disagree B. Somewhat disagree C. Neutral D. Somewhat agree E. Completely agree

4. If a platform recommends information that matches my interests, I am significantly more likely to click and view it than information on unfamiliar topics ( )

A. Completely disagree B. Somewhat disagree C. Neutral D. Somewhat agree E. Completely agree

5. Unless explicitly required for a task, I rarely actively search for or browse information in unfamiliar fields ( )

A. Completely disagree B. Somewhat disagree C. Neutral D. Somewhat agree E. Completely agree

6. I adjust the platform’s recommendation settings based on my information preferences (e.g., blocking certain content, following specific accounts) ( )

A. Not at all true B. Somewhat untrue C. Neutral D. Somewhat true E. Completely true

7. When friends or others share information unrelated to my interests, I usually do not read it in depth or seek further understanding ( )

A. Not at all true B. Somewhat untrue C. Neutral D. Somewhat true E. Completely true

III. Dimensions Related to Adler’s teleology

(1) Sense of Belonging

1. I am consistently active in one or more fixed online communities (e.g., fan groups, forum sections, gaming guilds) and feel a strong sense of belonging to them ( )

A. Not at all true B. Somewhat untrue C. Neutral D. Somewhat true E. Completely true

2. When my views receive widespread approval within my online community, I feel deeply reassured and satisfied ( )

A. Completely disagree B. Somewhat disagree C. Neutral D. Somewhat agree E. Completely agree

3. When I encounter information aligned with my interests, I experience a sense of belonging, thinking “I belong to this information circle.” ( )

A. Not at all true B. Somewhat untrue C. Neither agree nor disagree D. Somewhat true E. Completely true

4. To avoid conflicting with the prevailing views in my online community, I choose not to express or minimize expressing differing opinions. ( )

A. Not at all true B. Somewhat untrue C. Neutral D. Somewhat true E. Completely true

5. I actively follow trending topics widely discussed by others to find common ground for social interactions. ( )

A. Not at all true B. Somewhat untrue C. Neither true nor untrue D. Somewhat true E. Completely true

6. If a certain type of information helps me stay on the same page with important people around me, I’m more likely to actively seek it out ( )

A. Not at all true B. Somewhat untrue C. Neutral D. Somewhat true E. Completely true

(II) Sense of Control and Security

1. Faced with the vast and often contradictory information on the internet, I frequently feel confused and anxious ( )

A. Not at all true B. Somewhat untrue C. Neutral D. Somewhat true E. Completely true

2. I prefer topics and content with clear conclusions that align with my expectations, as this makes me feel in control ( )

A. Not at all true B. Somewhat untrue C. Neutral D. Somewhat true E. Completely true

3. I consciously unfollow or block accounts that frequently post information I find uncomfortable or difficult to agree with ( )

A. Not at all true B. Somewhat untrue C. Neutral D. Somewhat true E. Completely true

4. I tend to trust a few sources I consider “reliable,” which saves me the effort of sifting through chaotic information myself ( )

A. Not at all true B. Somewhat untrue C. Neither true nor untrue D. Somewhat true E. Completely true

5. When my preferred information platforms or accounts suddenly change their style or push content I find uninteresting, I feel uncomfortable ( )

A. Completely disagree B. Somewhat disagree C. Neutral D. Somewhat agree E. Completely agree

(III) Maintaining Self-Consistency

1. The information I believe is typically that which confirms and reinforces my existing ideas and values ( )

A. Completely disagree B. Somewhat disagree C. Neutral D. Somewhat agree E. Completely agree

2. When encountering compelling evidence that contradicts my core beliefs, my first instinct is to question the source or reliability of the evidence rather than my own beliefs. ( )

A. Completely disagree B. Somewhat disagree C. Neutral D. Somewhat agree E. Completely agree

3. Being exposed to too much negative information makes me doubt my own judgment, and this feeling is unpleasant ( )

A. Completely disagree B. Somewhat disagree C. Neutral D. Somewhat agree E. Completely agree

4. When views I endorse are widely disseminated as “truth,” I feel my value and wisdom are affirmed. ( )

A. Completely disagree B. Somewhat disagree C. Neutral D. Somewhat agree E. Completely agree

5. I tend to simplify complex moral or social issues into a binary opposition of “good vs. evil” or “right vs. wrong” because this aligns better with my existing cognitive framework ( )

A. Completely disagree B. Somewhat disagree C. Neutral D. Somewhat agree E. Completely agree

(IV) Sense of Superiority

1. I hope to accumulate deep knowledge in a specific field so that my expertise surpasses that of most people around me. ( )

A. Completely disagree B. Somewhat disagree C. Neutral D. Somewhat agree E. Completely agree

2. When I possess more detailed knowledge in specific information domains (e.g., gaming, cosmetics), I experience a sense of superiority thinking “I understand this better than others” ( )

A. Completely disagree B. Somewhat disagree C. Neutral D. Somewhat agree E. Completely agree

3. I tend to continuously deepen my expertise in information domains where I excel, highlighting my unique value by sharing insights within those fields ( )

A. Completely disagree B. Somewhat disagree C. Neutral D. Somewhat agree E. Completely agree

4. The information sources I follow make me feel “I’m constantly learning/improving,” even if the content is highly homogeneous. ( )

A. Completely disagree B. Somewhat disagree C. Neutral D. Somewhat agree E. Completely agree

5. I deliberately focus on certain types of information with barriers to entry (such as professional jargon or niche cultures) to distinguish myself from “ordinary information consumers.” ( )

A. Not at all applicable B. Not very applicable C. Somewhat applicable D. Fairly applicable E. Fully applicable

(V) Goal-Oriented

1. I proactively seek out specific types of information (e.g., graduate school preparation materials, workplace skills) to achieve clear goals in my studies, career, or personal life ( )

A. Completely disagree B. Somewhat disagree C. Neutral D. Somewhat agree E. Completely agree

2. Before seeking information, I first clarify my objectives (e.g., uncovering the truth about an event, learning a specific skill) and then filter corresponding information types ( )

A. Completely disagree B. Somewhat disagree C. Neutral D. Somewhat agree E. Completely agree

3. To efficiently achieve my goals, I focus on acquiring information directly relevant to them while ignoring irrelevant content.

A. Completely disagree B. Somewhat disagree C. Neutral D. Somewhat agree E. Completely agree

4. If a certain type of information does not support my goals (e.g., entertainment content when learning), I actively reduce the time spent on it ( )

A. Completely disagree B. Somewhat disagree C. Neutral D. Somewhat agree E. Completely agree

5. I adjust my information-seeking approach based on goal priority. ( )

A. Completely disagree B. Somewhat disagree C. Neutral D. Somewhat agree E. Completely agree

(VI) Avoiding Failure and Negative Emotions

1. I avoid information in unfamiliar fields (e.g., quantum physics, financial knowledge) because I fear I won’t understand it and will experience the frustration of “not being able to learn it.” ( )

A. Completely disagree B. Somewhat disagree C. Neutral D. Somewhat agree E. Completely agree

2. When confronted with information differing from my own views, I actively avoid it ( )

A. Completely disagree B. Somewhat disagree C. Neutral D. Somewhat agree E. Completely agree

3. I prefer to focus on familiar information because encountering new information makes me anxious about “uncertainty in mastering it” ( )

A. Not at all true B. Somewhat untrue C. Neutral D. Somewhat true E. Completely true

4. When encountering complex and unfamiliar information, I choose to avoid deeper understanding out of fear of not fully grasping it ( )

A. Not at all true B. Somewhat untrue C. Neither agree nor disagree D. Somewhat true E. Completely true

5. If certain types of information (e.g., negative news, controversial topics) may trigger irritation or anxiety in me, I will actively block such information. ( )

A. Completely disagree B. Somewhat disagree C. Neutral D. Somewhat agree E. Completely agree

6. I deliberately limit my information intake to specific areas to avoid the anxiety of “not knowing what to choose” when faced with overwhelming amounts of information. ( )

A. Not at all true B. Somewhat untrue C. Neutral D. Somewhat true E. Completely true

7. I avoid information that conflicts with my existing beliefs because I fear it might disrupt my cognitive habits and trigger negative feelings of self-doubt.

A. Completely disagree B. Somewhat disagree C. Neutral D. Somewhat agree E. Completely agree

Thank you once again for taking the time to complete the questionnaire! Your responses are crucial to the scientific validity of this study. Wishing you all the best!
