# Supplementary material for: The psychological motivation of users actively constructing information cocoons from the perspective of Adler’s teleology: an empirical study based on a sample of Chinese university students
Source: Front Psychol. 2026 Apr 24;17:1742055. doi: 10.3389/fpsyg.2026.1742055 (PMC13159072; doi:10.3389/fpsyg.2026.1742055)
Supplement: Supplementary file 1 [file Supplementary_File_1.zip › ▓╣│Σ▓─┴╧appendix/Survey Screening Questionnaire Chinese version.docx]

《从阿德勒目的论视角探究用户主动构建信息茧房的动机》调查筛选问卷

尊敬的受访者：​

您好！本问卷旨在探究用户对信息茧房概念理解的深度。请根据您对“信息茧房”概念的理解回答以下问题，这将帮助我们确保研究有效性。答案不影响参与资格，仅用于学术筛选。问卷填写约需3-5分钟，请您根据实际情况如实作答。感谢您的支持与配合！

1. 知识测试（共4题，每题1分）

1.信息茧房现象主要指：

A. 信息获取渠道的多样化

B. 只接触与自身观点相似的信息而形成封闭空间

C. 信息传播速度的加快

D. 信息质量的普遍提升

2.信息茧房的核心特征是：

A. 信息多样性 B. 信息同质化 C. 信息随机性 D.信息复杂性

3.算法推荐如何加剧信息茧房效应：

A. 通过扩大信息来源范围

B. 通过持续推送符合用户偏好的内容

C. 通过增加信息总量

D. 通过随机展示各种信息

4.信息茧房可能带来的主要影响是：

A. 增强批判性思维能力

B. 导致观点极化和认知局限

C. 提高信息处理效率

D. 促进社会共识形成

二、情景判断（共3题，每题1分）

1.“小明喜欢观看科技类视频，平台算法持续推荐相关内容，他很少接触到其他领域的信息。小明觉得这样很方便，能够快速获得感兴趣的内容。”请判断是否存在信息茧房？

是□ 否□

2.“小红每天会主动搜索不同观点的新闻，包括与自己立场相反的内容，以确保获得全面信息。”请判断是否存在信息茧房？

是□ 否□

3.“小张只关注自己认同的博主，逐渐听不到不同声音。”请判断这是否为信息茧房吗？

是□ 否□

三、自我效能检验（共3题，每题1分）

1.我能够清晰解释"信息茧房"的基本概念。

A.完全不符合 B.不太符合 C.一般 D.比较符合 E.完全符合

2.我能够识别出社交媒体中的信息茧房现象。

A.完全不符合 B.不太符合 C.一般 D.比较符合 E.完全符合

3.我了解信息茧房行为可能存在的影响。

A.完全不符合 B.不太符合 C.一般 D.比较符合 E.完全符合
