# Supplementary material for: The psychological motivation of users actively constructing information cocoons from the perspective of Adler’s teleology: an empirical study based on a sample of Chinese university students
Source: Front Psychol. 2026 Apr 24;17:1742055. doi: 10.3389/fpsyg.2026.1742055 (PMC13159072; doi:10.3389/fpsyg.2026.1742055)
Supplement: Supplementary file 1 [file Supplementary_File_1.zip › ▓╣│Σ▓─┴╧appendix/Survey Screening Questionnaire English version.docx]

Survey Screening Questionnaire

Dear Respondent,

Hello! This questionnaire aims to explore your understanding of the concept of the “information cocoon.” Please answer the following questions based on your understanding of the term “information cocoon.” This will help us ensure the validity of the research. Your answers will not affect your eligibility to participate and will only be used for academic screening purposes. The questionnaire will take approximately 3–5 minutes to complete. Please answer truthfully according to your actual situation. Thank you for your support and cooperation!

1. Knowledge Test (There are a total of 4 questions, each worth 1 point.)
2. The phenomenon of the information cocoon mainly refers to:

A. The diversification of information acquisition channels

B. Only being exposed to information that aligns with one’s own views, thereby forming a closed space

C. The acceleration of information dissemination speed

D. The general improvement in the quality of information

2. The core characteristic of the information cocoon is:

A. Information diversity

B. Information homogenization

C. Information randomness

D. Information complexity

3. How do algorithmic recommendations exacerbate the information cocoon effect?

A. By expanding the range of information sources

B. By continuously pushing content that aligns with user preferences

C. By increasing the total volume of information

D. By randomly displaying various types of information

4. The main potential impact of the information cocoon is:

A. Enhanced critical thinking ability

B. Leading to opinion polarization and cognitive limitations

C. Improved information processing efficiency

D. Promotion of social consensus

II. Scenario Judgment (There are a total of 3 questions, each worth 1 point.)

1. “Xiao Ming enjoys watching technology-related videos. The platform’ s algorithm continuously recommends similar content, and he rarely encounters information from other fields. Xiao Ming feels this is very convenient, as it allows him to quickly access content of interest.” Does this scenario indicate the presence of an information cocoon? □ Yes  □ No

2. “Xiao Hong actively searches for news with different viewpoints every day, including content that opposes her own stance, to ensure she receives comprehensive information.” Does this scenario indicate the presence of an information cocoon? □ Yes  □ No

3. “Xiao Zhang only follows bloggers he agrees with and gradually stops hearing differing opinions.” Does this an example of an information cocoon? □ Yes □ No

III. Self-Efficacy Check (There are a total of 3 questions, each worth 1 point.)

1. I am able to clearly explain the basic concept of the “information cocoon.”

A. Completely disagree B. Somewhat disagree C. Neutral D. Somewhat agree E. Completely agree

2. I am able to identify the phenomenon of information cocoons on social media.

A. Completely disagree B. Somewhat disagree C. Neutral D. Somewhat agree E. Completely agree

3. I understand the potential impacts that information cocoon behavior may have.

A. Completely disagree B. Somewhat disagree C. Neutral D. Somewhat agree E. Completely agree

Thank you once again for taking the time to complete the questionnaire! Your responses are crucial to the scientific validity of this study. Wishing you all the best!
